# Microemulsion Gels of Tapentadol Hydrochloride: Statistical Analysis of Pharmacokinetics and Skin Irritation Studies

**DOI:** 10.7150/ntno.110819

**Published:** 2025-05-07

**Authors:** Nimmathota Madhavi, Naveen Kumar Ganji, Heera Battu, Beeravelli Sudhakar

**Affiliations:** 1 Department of Pharmaceutics, CMR College of Pharmacy, Medchal, Kandlakoya, Hyderabad, Telangana 501401, India; 2 Department of Pharmaceutics, Brown's College of Pharmacy, Affiliated to Kakatiya University, Ammapalem, Khammam, Telangana 507305, India; 3 Adikavi Nannaya University College of Pharmaceutical Sciences, Tadepalligudem, Andhra Pradesh 533296, India; 4 Department of Pharmaceutical Technology, AU College of Pharmaceutical Science, Andhra University, Visakhapatnam, Andhra Pradesh 530003, India

**Keywords:** Microemulsion, Pharmacokinetics, Correlation, Dermal delivery, Skin irritation.

## Abstract

**Background:** The present study aimed to overcome the drawbacks of tapentadol through the oral route and to assess the significance of microemulsion gels for transdermal delivery via pharmacokinetic approach.

**Methods:** Microemulsions were prepared via a ternary phase diagram. The optimized microemulsion was converted into gels, and the microemulsion was evaluated for particle size, zeta potential and cumulative *in vitro* drug release, whereas the gel was characterized for viscosity, spreadability, *in vitro*, *ex vivo*, *in vivo* and skin irritation studies. The prepared ME-gel PKs were tested against MEs, oral solution and plain gel.

**Results:** The PK study revealed that the half-life of the ME gel was 2.2-fold greater than that of the oral solution and 1.65-fold greater than that of the plain gel. The MRT of the ME gel was 6-fold greater than that of the oral solution and 3.3-fold greater than that of the plain gel. The overall mean value of the AUC (0-∞) was 3.16 times greater than that of the oral route. The skin irritation studies found that absence of irritation and damage after application of ME-gel.

**Conclusion:** The PK study revealed that ME-gel was effective in pain management. The level A IVIVC between the *in vitro* fraction of drug released and the fraction of drug absorbed *in vivo* was 0.9731.

## Introduction

Tapentadol hydrochloride (TPHCl) is a centrally acting μ-opioid receptor that is used as an analgesic, preferably in the treatment of acute pain, chronic pain, neuropathic pain and pain with an inflammatory origin. Moreover, it may undergo rapid first-pass metabolism, requires frequent administration and has a half-life of only 4 h of elimination owing to its oral limitations; the current research aimed to develop an alternative route of drug delivery of TPHCl 1-2. An alternative drug delivery route to the oral route may be described as a transdermal drug delivery system (TDDS). It offers a steady-state concentration of drugs through the skin through an extensive period of time through controlled delivery 3-4. The drug or active medication must penetrate deeper layers of the skin through the upper barrier layer, i.e., the stratum corneum (SC), and the drug can enter the systemic circulation through the diffusion process 5. In TDDSs, a challenging aspect of the dosage form is to improve permeation through various excipients that are supported to form a dosage form. These systems include many nanolipid carrier systems, such as liposomes, niosomes, transferosome microemulsions and microneedles 6-7. Among the above dosage forms, the present research focused on developing microemulsions (MEs) because of their benefits. MEs are clear, thermodynamically stable, isotropic liquid mixtures of oil, water and surfactants/cosurfactants 8. They are capable of encompassing both hydrophilic and lipophilic drugs 9. MEs can serve as rate-limiting membrane barriers and, upon their systemic absorption, are capable of acting as controlled drug delivery systems. Hence, the aim of our current research was to develop a transdermal ME-loaded gel of TPHCl that succeeds in ensuring the presence of TPHCl at the required steady-state concentration of plasma over a prolonged period of time. Compared with oral gels, transdermal gels usually contain a small percentage of drugs. These gels promote the absorption of medication through the skin. When gel is applied to the skin, the drug is absorbed directly into the blood stream to work at a systemic level 10-11.

PK parameters are crucial in formulation development for existing drugs because *in vitro* tests cannot anticipate *in vivo* performance, which is much required to analyze the therapeutic efficacy of the drug product 12-13. In addition, PK studies have explored the therapeutic drug concentration in plasma to reduce the incidence of side effects 14. Hence, the present work aims to develop ME-gels and perform various studies, such as *in vitro*, *ex vivo* and *in vivo* studies. Furthermore, this study compared the *in vitro*-*in vivo* correlation (IVIVC) of the test product with other collate dosage forms and the correlation coefficient. Therefore, this research appraises the potential therapeutic efficacy of the prepared ME-gel.

## Materials and Methods

### Materials

TPHCl was obtained as a gift sample from MSN laboratories Pvt Ltd., Hyderabad, India. Capryol 90, Labrasol, and transcutol P were obtained as gift samples from BASF Pvt Ltd., and carbopol 934 NF was purchased from Merck Pvt Ltd. Mumbai, India. All other chemicals used in the study were of analytical grade and were obtained from SD Fine Chemicals, Mumbai, India.

### Construction of pseudoternary phase diagrams

Pseudoternary phase diagrams comprising an oil, a surfactant and a cosurfactant were constructed via the aqueous titration method at ambient temperature. Capryol 90 was selected as the oil phase, labrasol was used as the surfactant, and transcutol P was used as the cosurfactant. The ratio of surfactant to cosurfactant (Smix) was varied at ratios of 1:1, 1:2, 1:3, 2:1 and 3:1, and the ratios of oil to cosurfactant (Smix) were 9:1, 8:2, 7:3, 6:4, 5:5, 4:6, 3:7, 2:8 and 1:9 (w/w). The end point of the titration appeared as a clear solution or was sometimes cloudy or turbid. The aqueous phase was added to make a mixture clear, and the pseudoternary phase diagrams were plotted via Tri-plot software version 4.1.2. (Todd Thompson software) 15-16.

### Formulation of MEs and their gels

From the obtained results of the pseudoternary phase diagrams, the highest ME region was found to be 3:1. Accurately weighed amounts of the pure drug, oil phase, surfactant and cosurfactant were added together, and the system was stirred with a magnetic stirrer at 400 rpm at room temperature (25°C) until a uniform distribution of the drug was achieved 17. On the basis of the physicochemical evaluation, ME5 was optimized, and various concentrations of the gels were prepared by using Carbopol 934 NF as a gel base 18. Triethanolamine was used to neutralize and adjust the pH of the gel to 7.0, and the formulation was left at room temperature (25±3°C) for 24 hrs to hydrate. On the basis of the gel characterization parameters, the selective concentration of the ME-gel was optimized, and it can be used for further evaluation.

### Characterization of Gels

#### Determination of Viscosity

The gel samples were placed on a viscometer plate and analyzed for viscosity (η), shear stress (τ) and shear rate at various speeds and at 100 rpm. The gels of various concentrations (1%, 1.5% and 2%) were subjected to rheological studies, which were performed by using a Brookfield cone and plate rheometer model LV-DV III. The viscosity properties of the test formulations were studied at 25±0.1°C for 1, 2 and 3% w/v concentrations of test products 16.

#### Determination of spreadability

The spreadability of the gel formulations was determined by using wooden block and glass slide apparatus. The apparatus consists of a wooden block with a fixed glass slide and a movable glass slide with one end tied to a weight pan rolled on the pulley. One gram of the test formulation was spread on a fixed glass slide. Another slide was placed over this sample, which was attached to a fixed load weight of 100g for 5 min, after which the weight was removed from the upper slide, and the spreadability was measured in terms of the time in seconds taken up by the two slides to slip off the gel 17. The spreadability of the gel was calculated via equation (1):



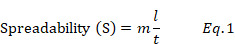



where m is the weight of the gel, l is the length and t is the time.

### *In vitro* drug release and *ex vivo* permeation studies

The *in vitro* drug release studies were performed by using a Franz diffusion cell with a diffusional area of 4.52 cm2. Test samples (pure TPHCl solution, plain gel and ME-gel) equivalent to 5 mg were used for the study. A cellulose dialyzing membrane (Membra-Cel MD 34-14, cutoff of 14 kDa) was used as the barrier for *in vitro* drug release studies. A weight equivalent to 5 mg of test formulation was placed in the donor compartment, and 25 ml of phosphate buffer (pH 7.4) was used as the receptor medium. The experimental system was maintained at 32°C±0.5°C with a magnetic stirrer at 100 rpm. At predetermined time intervals (1, 2, 4, 6, 8, 10, 12, and 24 h), one mL aliquots were withdrawn from the sampling port and replaced with an equal volume of fresh buffer. The samples were analyzed spectrophotometrically at 272 nm. For *ex vivo* permeation studies, Albino (Wistar strain) rat skin was used instead of a dialyzing membrane, and the test formulation was applied in the position of excised rat skin with the SC facing upward into the donor compartment 16-17. The remaining procedure was the same as that of the *in vitro* studies.

### Drug and excipient interaction studies by FTIR

The IR spectra of the samples were recorded on a Bruker FTIR spectrophotometer equipped with Opus software. The test samples were subjected to ATIR. Appropriate amounts of TPHCl, labrasol, transcutol p, ME5 and ME-gel were used. The IR spectra of the drug, excipients and formulation samples were recorded in the range of 400-4000 cm-1 17.

### PK Study

The *in vivo* study protocol was reviewed and approved by the Institutional Animal Ethics Committee of A.U. College of Pharmaceutical Sciences, Andhra University (Reg. No. 516/PO/c/01/IAEC/7). The *in vivo* studies were carried out on albino Wistar male rats weighing approximately 200-250 g. After the rats were procured from the central animal house facility to the laboratory, they were kept in cages in the laboratory and acclimatized to the laboratory surroundings and to room temperature (25±3°C) for a period of one week.

### Experimental design

The abdominal area of each rat was shaved just before the experiment, and the rats were kept under observation for 24 hrs for any skin irritation that may have occurred during shaving. The animals were divided into four groups, and each group contained three animals. The test products of the ME-gel, ME and plain gel were applied transdermally, whereas the TPHCl solution was applied orally. A dose equivalent to 5 mg/kg was used for the study. The four groups of animals were administered various doses in the following manner. The Group I animals were administered oral solution, the Group II animals were treated with ME-gel, the Group III animals were treated with ME, and the Group IV animals were treated with plain gel. The selected products were applied on the dorsal side of the abdominal skin of the rats. A porous gauge dressing and nonirritating tape (3 M transpore) were attached to the skin on top of the application to ensure that it was not removed from its site of application 17-19.

### Sample collection

Samples of 300-µL aliquots of blood were collected from the rat retro-orbital sinus into microcentrifuge tubes containing dipotassium ethylene diamine tetra acetic acid. Samples were collected periodically at 0, 0.5, 1, 2, 3, 4, 6, 8, 10, 12 and 24 hrs. The plasma was immediately separated by ultracentrifugation under refrigeration (5°C) at 5000 rpm for 15 min, and each plasma sample was collected and stored at -20°C until drug analysis.

### Data analysis

Non-compartmental analysis of plasma data after extravascular input was evaluated via PK Solver 2.0 (MS Excel add-in). PK parameters such as the area under the curve (AUC_0-∞_), elimination rate constant (K_el_), elimination half-life (t_1/2_), area under the first moment of the plasma concentration‒time curve (AUMC) and mean residence time (MRT0--24) of TPHCl after different treatments were calculated.

### Analytical methods

The plasma concentration of TPHCl was determined via a modified RP-HPLC method. A mixture of 20 mM sodium perchlorate buffer (pH 6.8) and methanol (40:60 v/v) was used as the mobile phase at a flow rate of 1 mL/min by using an Altima Grace Smart C-18 column. The mixture was sonicated for 40 min before use and filtered through a 0.45-μm PVDF membrane filter. TPHCl was detected at 236 nm with a retention time of 4.78 min. In the present study, the analytical method was validated according to standard procedures 20. The linearity ranged from 50-300mg/mL. The coefficients of variation for the intra- and interday precision were < 3.03%. The intra- and interday accuracies were 98.28-102.99%. The limit of quantification (LOQ) for TPHCl was 50 mg/ml.

### Relative bioavailability (Frel)

Relative bioavailability refers to the availability of a drug product compared with another dosage form or product of the same drug given at the same dose.



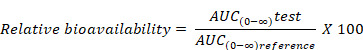



### *In vitro*-*in vivo* correlation (IVIVC)

The results obtained through *in vitro* and *in vivo* studies were IVIVC via the deconvolution method. IVIVC is a point‒to‒point correlation between the *in vitro* fraction of drug released and the fraction of drug absorbed *in vivo*. The amount absorbed is not measured directly but rather obtained through deconvolution. The level A correlation is supposed to be the best variety of correlations 21.

### PK and statistical analysis

The PK parameters were statistically analyzed via Prism version 5.0 software (GraphPad Inc., USA) and calculated via a non-compartmental approach via PK solver software. The maximum plasma concentration (C_max_), maximum time to reach peak plasma concentration (T_max_), first-order elimination rate constant (K_el_), elimination half-life (t_1/2_), and mean residence time (MRT_0-t_) were used. The relationships and variances between the PK parameters were statistically evaluated via t-tests. All the values were compared with those of the plain gel and are expressed as the mean±SD, and analysis of variance (ANOVA) was performed at a significance level of 0.05, which was used for comparison of the PK parameters.

### Skin Irritation studies

Using albino Wistar rats, the modified Draize test was used to assess primary cutaneous irritation investigations. We carried out skin irritation experiments to evaluate the potential irritant effects of ME-gel. Acute cutaneous irritation investigations were used to evaluate these in accordance with OECD criteria. The Animal Ethical Committee of the A.U. College of Pharmaceutical Sciences authorized the experimental procedure (Reg. No. 516/PO/c/01/IAEC/7). For the investigation, male Wistar rats weighing between 200 and 250 g were employed. Before the trial, the animals were acclimated for at least seven days. Before application of test formulation, rat's dorsal skin surface was completely shaved without hair. The test formulation was uniformly placed over the rats' skin in the amount of half a gram. Non-sensitizing micropore tape was used to cover the application location. After the formulations were applied, the skin was checked for any obvious changes, such as erythema (redness) and edema, 24 and 48 hours later 22-23.

## Results

A stable microemulsion was formed with the right blend of low and high HLB (hydrophilic lipophilic balance) surfactants. On the basis of the solubility data, capryol 90, labrasol and transcutol P were selected as the oils, surfactants, and cosurfactants, respectively, and were used to construct the pseudoternary phase diagram. The concentrations of the components were recorded via Tri-plot software version 4.1.2 (Todd Thompson software). The total mixture composition of the triangle represents 100% w/w. The systems providing more ME regions from the four component systems were identified, and a 3:1 ratio was selected.

### Characterization of gels

#### Viscosity of gels

The average viscosity of 1% w/v was the lowest (1322.62 Cps) among the three prepared gels (1.5% w/w viscosity 1529.28 Cps and 2% w/w 2260.37 Cps). Thus, the 1% w/w gel mixture was optimized on the basis of having the lowest average viscosity.

#### Determination of spreadability

The spreadability of the plain gel and ME-gel was 48.2±0.2 and 53.7±0.3 gm.cm/s, respectively. This shows that the ME-gel has good spreadability compared with the plain gel.

### *In vitro* and *ex vivo* permeation studies

The *in vitro* permeation rates were 92.2±0.5% and 76.4±0.1% in 24 h for the ME-gel and plain gel, respectively, up to 24 h. The flux values were 0.0425±0.2 and 0.0352±0.6 mg/cm^2^/hr for the ME-gel and plain gel, respectively. The permeability coefficient (Kp) value of the ME-gel was 8.5x10^-3,^ whereas that of the plain gel was 6.5x10^-3^. Drug release from the gel formulations followed a first-order mechanism of drug release followed by non-Fickian diffusion, as indicated by 'n' values of 0.562 and 0.538 for the ME-gel and plain gel, respectively. The *ex vivo* cumulative percentage permeation values were 88.6±0.1% and 62.4±0.3% at 24 h for the ME-gel and plain gel, respectively. The flux (mg/cm^2^/hr) values were 0.0281±0.7 and 0.0408±1.8 for the plain gel and ME-gel, respectively. The permeability coefficient (Kp) values of the ME-gel and plain gel were 8.1x10^-3^ and 5.6x10^-3^, respectively. Drug release followed non-Fickian diffusion, as indicated by 'n' values of 0.612 and 0.845 for the ME-gel and plain gel, respectively.

### FTIR studies

**Figure 3:**
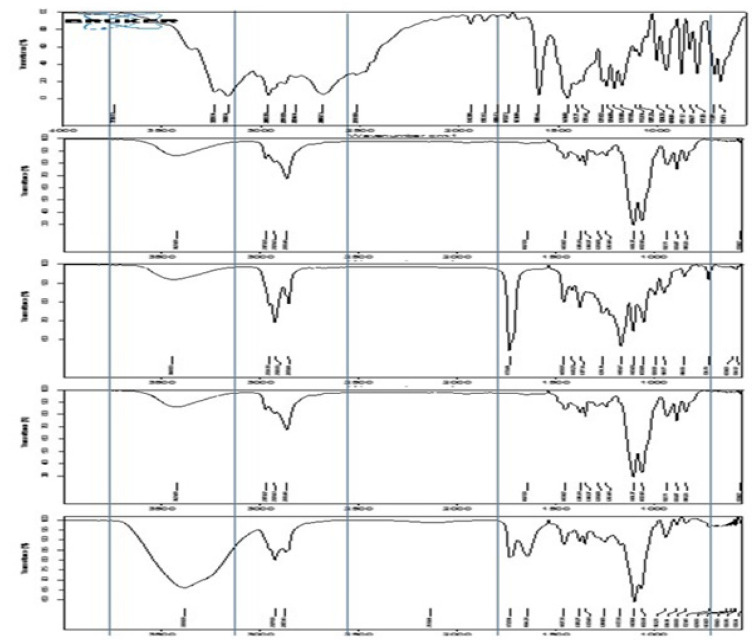
FTIR Spectrum overlay of TPHCl, Transcutol p, Labrasol, 3:1 ratio of microemulsion and ME gel.

As shown in Figure 3, the infrared spectra of TPHCl, transcutol p, labrasol, and the 3:1 ratio of ME to ME-gel present characteristic peaks.

### *In vivo* pharmacokinetic study

**Figure 4:**
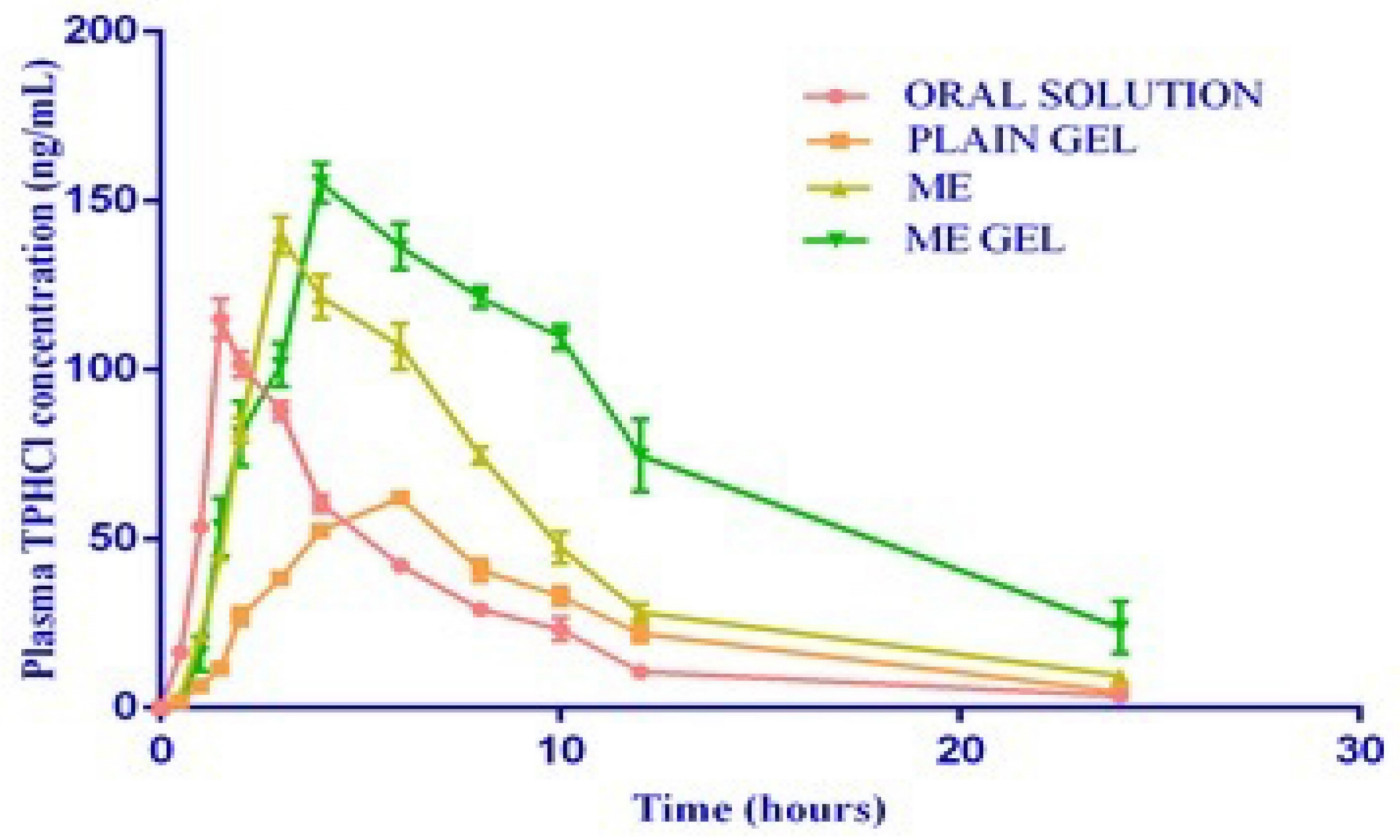
Mean plasma concentrations Vs time profiles of test products.

The test formulations (oral solution, microemulsion-based gel, drug-loaded plain gel, and microemulsion) were very well tolerated by healthy rat subjects. No adverse reactions, such as gastrointestinal disturbances or allergic reactions, were observed during the study period. The complete PKs of the test formulations are shown in Figure 4.

The mean K_el_ values of the test formulations were 0.16±0.15, 0.14±0.01, 0.13±0.01 and 0.11±0.01 h^-1^ for the oral solution, plain gel, ME and ME gel, respectively. A bar diagram of the K_el_ of the test products is shown in Figure 3. The mean K_el_ values are observed to be in a narrow range of 0.11-0.16. The values are tabulated in Table 1.

**Figure 5:**
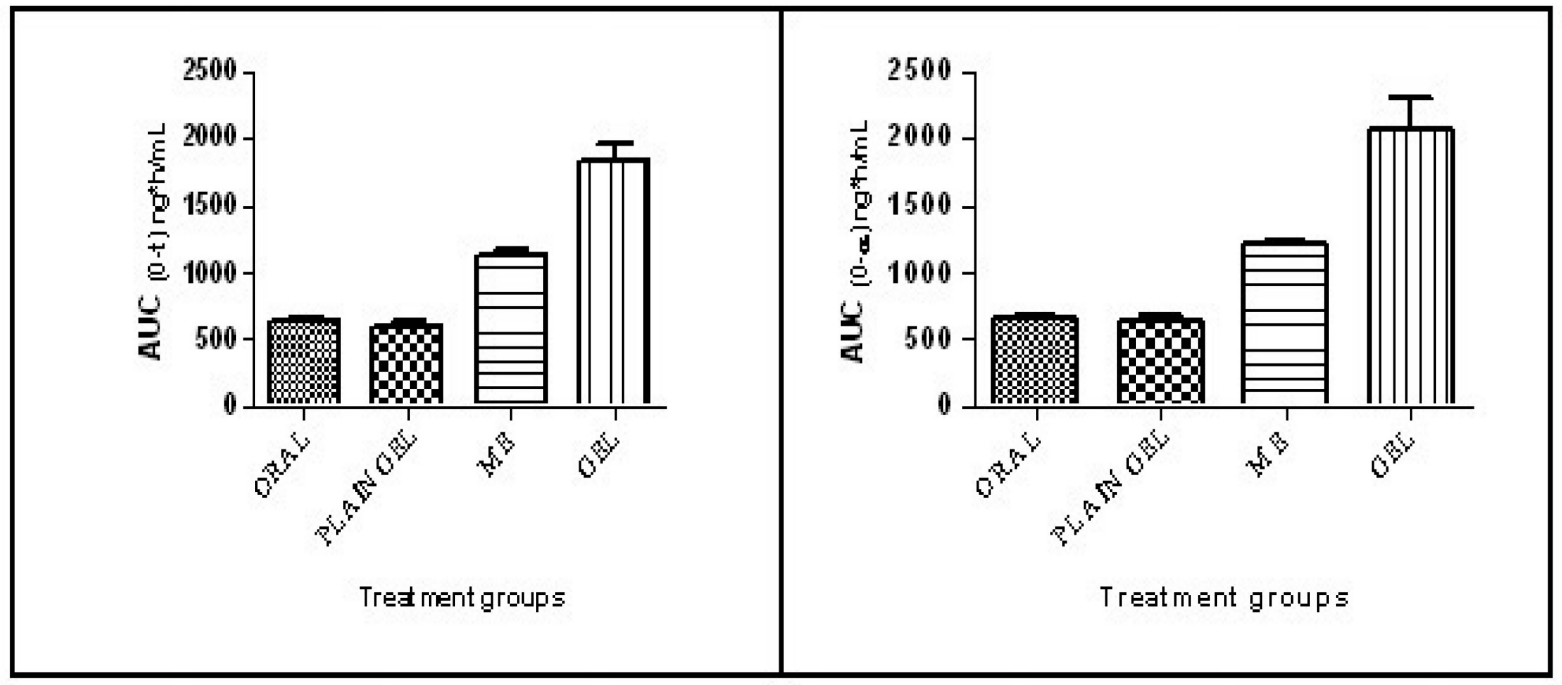
*AUC_0-t_* and *AUC_0-∞_* in different treatment groups.

The mean half-life (t_1/2_) values of the test formulations were found to be 4.51±0.2,5.09±0.40, 5.23±0.26 and 6.74±1.44 h for the oral solution, plain gel, ME and ME-gel, respectively. The T_max_ values of the test formulations were found to be 1.5, 6.0, 3.0 & 4.0 h for the oral solution, plain gel, ME and ME-gel, respectively. The values are shown in Table 1. The mean C_max_ values of the test formulations were found to be 114.93±5.89, 62.13±2.08, 139.75±5.61 and 155.30±5.78 ng/mL for the oral solution, plain gel, ME and ME-gel, respectively. The values are shown in Table 1. The C_max_ values are tabulated in Table 1. The mean AUC_0-24_ values of the test formulations were 632.91±25.28, 601.27±41.45, 1131.80±35.11 and 1831.17± 26.15 ng/mL h for the oral solution, plain gel, ME gel and MEgel, respectively. The results are illustrated in Table 1. The mean AUC_0-∞_ values of the test formulations were 656.08±25.49, 635.64 ±40.56, 1204.22±33.36 and 2072.89±233.87 ng/mLh for the oral solution, plain gel, ME gel and MEgel, respectively. The values are illustrated in Table 1. The AUC_0-24_ values are shown in Figure 5.

The mean AUMC_0-∞_ values of the test formulations were found to be 4452.79±202.60, 6140.27±408.99, 10778.08±549.42 and 25199.51±6393.51 ng/mL. h^2^ for the oral solution, plain gel, ME gel and MEgel, respectively. The mean MRT values of the test formulations were found to be 6.79±0.18, 9.66±0.33, 8.95±0.38 and 12.03±1.81 hrs for the oral solution, plain gel, ME and ME-gel, respectively. The values are illustrated in Table 1.

### IVIVC

**Figure 6:**
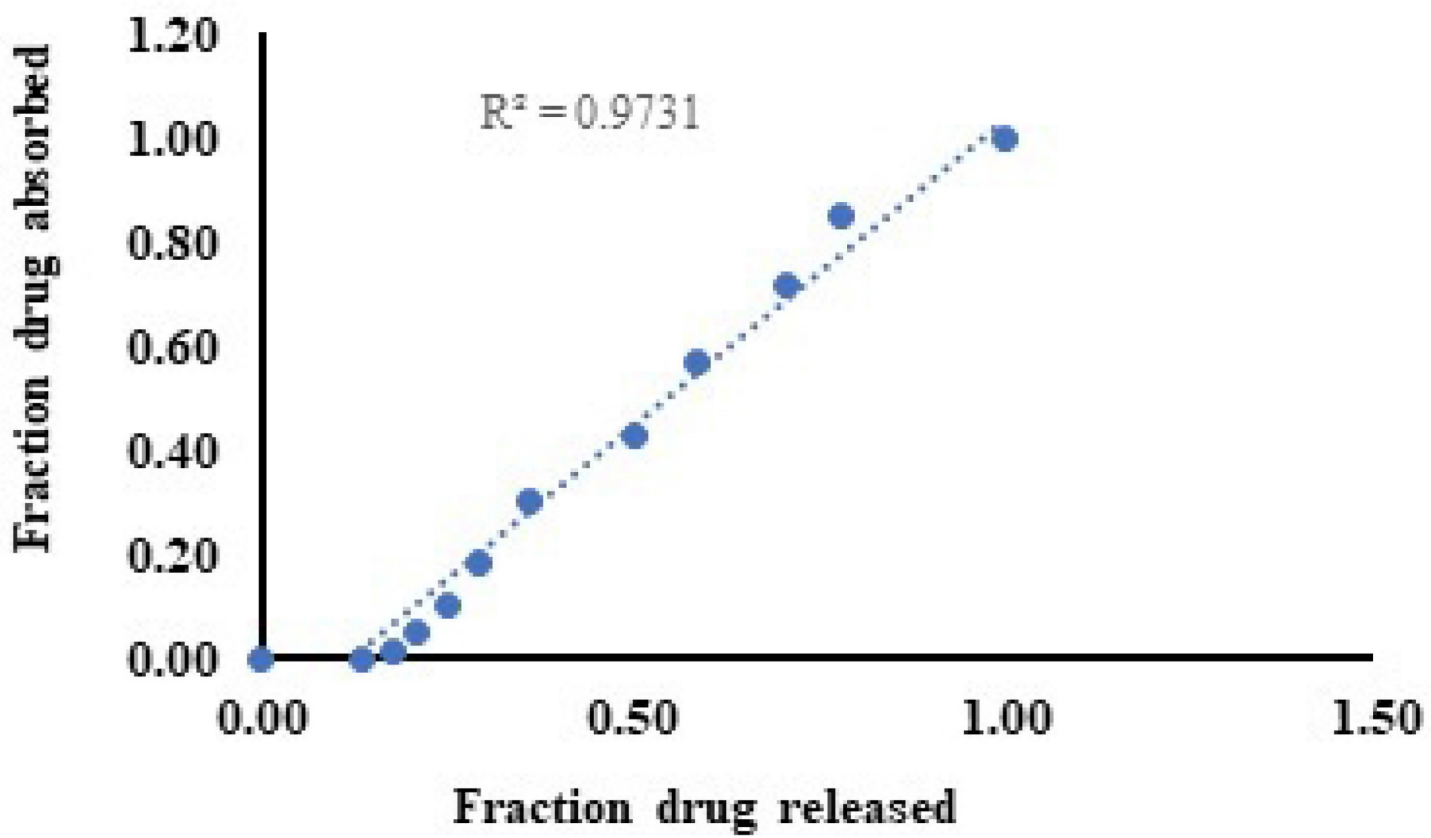
Correlation between percentage fraction drug released and fraction drug absorbed.

The level A IVIVC is a point-to-point correlation between the *in vitro* fraction of drug released and the fraction of drug absorbed *in vivo,* as shown in Table 2. The amount absorbed is not measured directly but rather obtained though deconvolution and the observed correlation was shown in Figure 6.

### Skin irritation studies

The skin irritation studies could not find any irritation or erythema indicating that the ME-gel were non-irritant and safe. The excipients used in formulation are safe for topical drug delivery. In the photograph we clearly observed that there is no damage or irritation even after 48 hrs of gel application.

## Discussion

The microemulsion gels were optimized based on several parameters results because the developed formulation is meant for skin delivery. Hence the major parameters evaluation as discussed**.** Form the viscosity results, the 1% w/w gel mixture was optimized on the basis of having the lowest average viscosity. The higher the viscosity of the gel is, the slower the drug release. A low viscosity of the gel is required at the site of application to allow better penetration of the oil globules of the optimized ME-gel into the skin and it was well correlated with earlier research of Lisa Binder *et al*., 2019 24. The lower viscosity of the ME-gel also led to a short residence time on the skin surface. Viscosity also affects the spreadability of the ME-gel. Hence, a formulation with optimum viscosity was selected. The ME-gel has good spreadability compared with the plain gel. This could be because of the loose gel matrix of the ME-gel due to the presence of oil globules rather than the plain gel formulation. From the *in vitro* studies, the pattern of drug release observed initially slowed and then gradually increased up to 24 h. The maximum flux was obtained from the ME-gel, which indicated the highest degree of drug permeation. Whereas form *ex vivo* studies, the highest flux from the ME-gel resulted in the highest degree of drug permeation through the rat skin.

The *in vitro* and *ex vivo* permeation studies were interpreted in the following manner. Compared with the plain gel, the ME-gel shows the highest percentage of permeation. The particle size of the ME-gel was approximately 126.6±0.1 nm, which helps increase permeation. As per the literature Yi-Bin Fan *et al*., 2021 25, stated that the particle size of 300 nm penetrates deeper into the skin layer and even 30nm is also promising limitation for skin drug delivery. In the current study attained desirable particle size which is suitable for skin drug delivery. ME-gel altered the physicochemical properties of the skin and improved permeation, whereas plain gel could not cross the dermal barriers, possibly because of its large particle size and low spreading efficiency. Flux was found to be proportional to the gradient of thermodynamic activity rather than the concentration. The thermodynamic activity of the drug decreases as the solubility in the solvent increases. During *ex vivo* permeation, the ME-gel interacts with the skin lipids, and a lipophilic nanosized emulsion may enhance the thermodynamic activity of the drug and thereby increase the permeation rate and flux across the skin. The FTIR results show prominent peaks for the active groups present in TPHCl. These studies revealed compatibility between the drug and excipients.

### *In vivo* pharmacokinetic study

The mean K_el_ value of the ME-gel was found to be the lowest, and the oral solution had the highest value. The elevated K_el_ values observed may be due to the permeation of the drug from the ME-gel into the vascular system, as the drug is entrapped or enclosed in nanosized micelles; hence, it escapes elimination in organs for long periods. The results of ANOVA and multiple comparison tests revealed that there was no significant difference in the K_el_ or t_1/2_ between the oral solution and plain gel, as the drug was delivered as such. However, in the different ME and ME-gel treatment groups, there was a significant difference in the K_el_ and t_1/2_ compared with those of the oral solution and plain gel, as the drug is formulated as an ME, which delays drug elimination from the body. When administered as an oral solution, TPHCl rapidly absorbs and attains C_max_ within 1.5 hrs. However, when administered as a plain gel transdermally, the drug takes a longer time to be absorbed, as the drug must pass through the skin layers; hence, the T_max_ is greater. However, when the drug is formulated as an ME, rapid and enhanced permeation of the drug through the skin layers can be observed, resulting in a lower T_max_ than that of the plain gel.

In all the treatment groups, lower C_max_ levels were detected in the plain gel-administered group, whereas higher C_max_ levels were detected in the ME-gel-treated group. The results of ANOVA and multiple comparison tests indicated that there was a significant difference among the different treatment groups. Compared with that of the oral solution, the C_max_ of TPHCl was 1.22- and 1.35-fold greater when TPHCl was administered as the ME and ME-gel, respectively. This can be attributed to the first-pass metabolism experienced by the drug when it is administered orally. Compared with that of the plain gel, the C_max_ of TPHCl was 2.24 and 2.49 fold greater when it was administered as ME and ME-gel, respectively, which can be attributed to enhanced permeation when it was formulated as ME. The C_max_ values were lower in the plain gel administered group, whereas higher C_max_ values were observed in the ME-gel treated group, which may have been due to the first-pass metabolism experienced by the drug when it was administered orally. Among all the treatment groups, the lowest AUC_0-24_values were shown by the plain gel group, whereas the highest AUC_0-24_values were shown by the ME-gel-treated group. The results of the ANOVA and multiple comparison tests indicated that there were significant differences among the different treatment groups. Among all the treatment groups, the lowest AUC_0-∞_values were shown by the plain gel group, whereas the highest AUC_0-∞_values were shown by the ME-gel-treated group. The results of the ANOVA and multiple comparison tests indicated that there were significant differences among the different treatment groups.

The AUC_0-24_ of TPHCl in ME and ME-gel was found to be 1.81- and 2.93-fold greater than that of TPHCl from oral solution and 1.88- and 3.05-fold greater than that of the plain gel, respectively, which indicated improved bioavailability when TPHCl was administered transdermally to avoid first-pass metabolism. The AUC_0-∞_ of TPHCl in ME and ME-gel was found to be 1.84- and 3.16-fold greater than that of the oral solution and 1.89 and 3.26 fold greater than that of TPHCl from the plain gel, which indicated the improved transdermal bioavailability of TPHCl by ME. Taken together, these findings confirmed that TPHCl, when formulated as MEgel and administered transdermally, can promote drug permeation via the skin after *in vivo* administration and improve bioavailability. One of the research study revealed that in an *in vivo* PK study in Wistar rats, microemulsion achieved around two folds higher bioavailability than pure drug solution 26. The result was similar to our present research work.

The lowest AUC_0-24_ values were shown by the plain gel group, whereas the highest AUC_0-24_ values were shown by the ME-gel-treated group, indicating improved bioavailability when the drug was administered transdermally. Compared with those of the oral solution, the increased MRT values of the ME-gel samples indicated that the effective plasma concentrations were maintained for longer time periods than those of the oral solution. Compared with the oral solution, the ME-gel resulted in a 1.77-fold increase in the MRT, indicating that the effective plasma concentrations were maintained for longer time periods 27.

### IVIVC

In the case of transdermal delivery, the *in vitro* property is the rate of permeation through the skin, whereas the *in vivo* response is the plasma drug concentration. For a transdermal product, the passage of the drug across the SC, rather than release from a transdermal formulation, is the slowest step in drug transport from the dosage form through the skin into the systemic circulation. The barrier of the SC is typically well preserved in excised skin, and consequently, the rate-limiting step in *in vitro* drug transport corresponds well to the absorption rate-limiting step *in vivo*. A level IVIVC is a point-to-point correlation between the *in vitro* fraction of drug released and the fraction of drug absorbed *in vivo*. The amount absorbed is not measured directly but rather obtained though deconvolution.

The level A correlation is supposed to be the best variety of correlations. This model establishes a linear relationship between *in vitro* drug release and *in vivo* drug permeation, and the level-A correlation was observed. A good correlation coefficient value of 0.9731 was observed between the fraction of drug released *in vitro* and the fraction of drug absorbed *in vivo,* and a linear relationship was observed between the two variables 28.

### Skin irritation studies

The positive result of skin irritation is may be because of lack of irritation is due to the reason that the surfactant and co surfactant used in the formulations are chosen from the non-ionic group of surfactants, which show the property of lower irritation and good cutaneous tolerance. In ME-gel, increased viscosity and three-dimensional network structure of carbopol 934NF reduced the chances of direct contact of microemulsion with the skin layers 29-31.

## Conclusion

The results of the present study were consistent with the PK results of microemulsion gels for dermal delivery. The dosage forms were developed on the basis of pseudoternary phase diagrams. The optimized ME was formulated into an ME gel, and its efficiency was calculated through various parameters. The results from the *in vivo* PK study revealed that, compared with other test formulations, TPHCl permeated well from the ME-gel via the transdermal route. The K_el_, t_1/2_, C_max_, T_max_ and AUC profiles were compared. When all the treatment groups were compared, the C_max_ was greater for the transdermal route than for the oral route. This occurred because TPHCl is metabolized first by passes when it is administered transdermally. The T_max_ values were greater for the groups that received transdermal administration than for the groups that received oral administration; this difference might be attributed to the fact that SC could delay the permeation of TPHCl from ME-gels in contrast solution administered orally, which is an immediate release dosage form. The overall mean value of AUC_0-∞_obtained via the transdermal route was 3.16 times greater than that obtained via the oral route, demonstrating that the improved bioavailability of TPHCl is probably from the ME-gel. This could be due to the avoidance of first-pass hepatic metabolism via the transdermal route. Therefore, TPHCl in the form of ME-gel could be an ideal dosage for pain management.

## Figures and Tables

**Figure 1:**
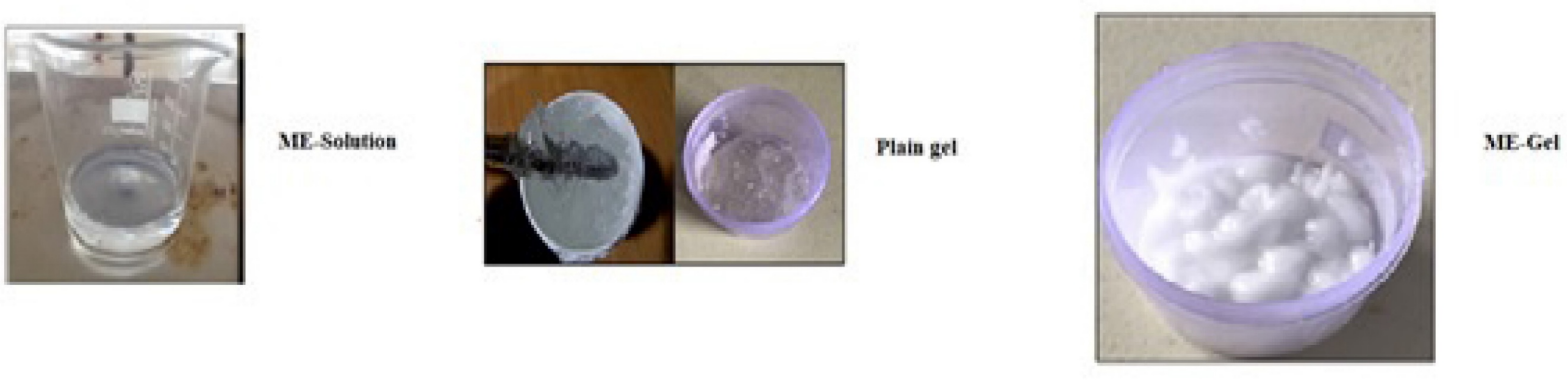
Various test formulations.

**Figure 2:**
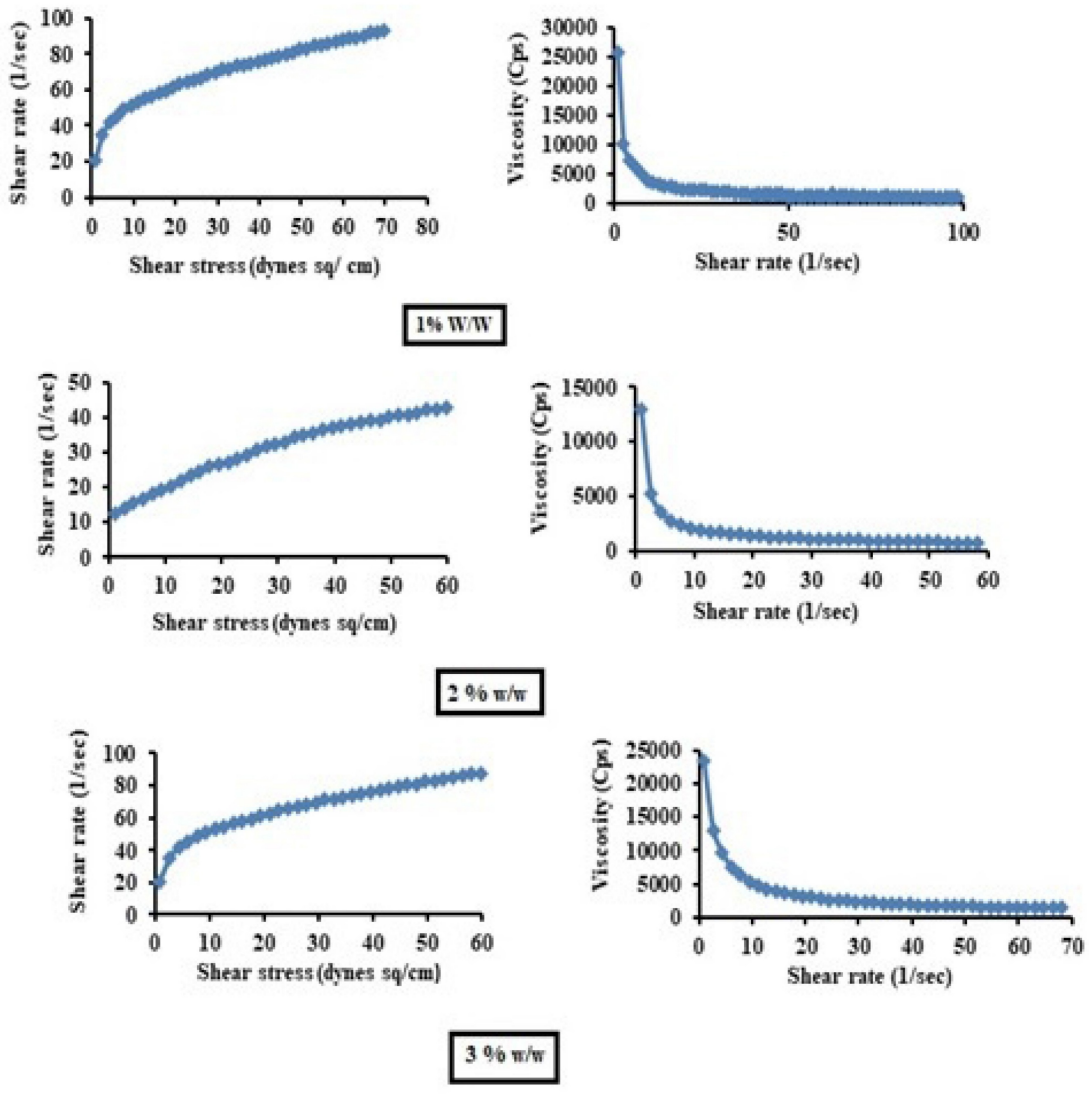
Viscosity profiles of various concentrations of test gels.

**Figure 7:**
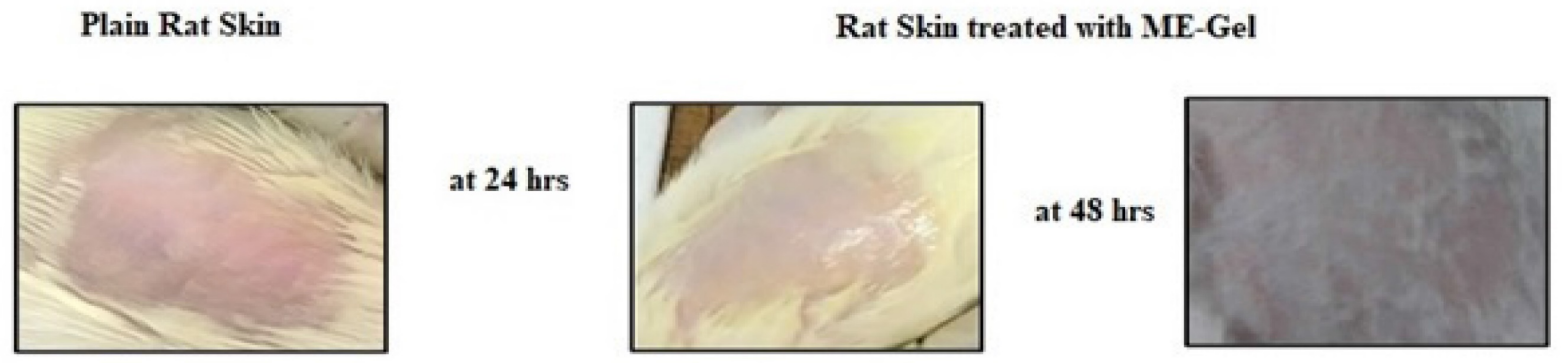
Skin Irritation studies of selected ME-Gel.

**Table 1:** Pharmacokinetic parameters of TPHCl test products

Parameters	Oral solution	Plain Gel	ME	ME Gel
K_el_(h-1)	0.16±0.15	0.14±0.01	0.13±0.01*	0.11±0.02*#
t_1/2_ (h)	4.51±0.83	5.09±0.40	5.23±0.26*	6.74±1.44*#
T_max_ (h)	1.50±0.83	6.00±0.61	3.0±0.98	4.00±0.93
C_max_ (ng/mL)	114.93±5.89#	62.13±2.08*	139.75±5.61*#	155.30±5.78*#
AUC_0-24_(ng/mL*h)	632.91±25.28	601.27±41.45	1131.80±35.11*#	1831.17±126.15*#
AUC_0-∞_(ng/mL*h)	656.08±25.49	635.64±40.56	1204.22±33.36*#	2072.89±233.87*#
AUMC_0-∞_(ng*/mL*h^2^)	4452.79#	6140.27±408.99*	10778.08±549.42*#	25199.51±6393.51*#
MRT (h)	6.79±0.18#	9.66±0.33*	8.59±0.38*#	12.03±1.81*#

*P<0.05, with Group I; # P<0.05, with Group II

**Table 2:** Fraction of drug absorbed from ME gel formulation

Time (h)	Ct (ng/mL)	(AUC)t_n-1_ -t_n_ (ng.h/mL)	(AUC) 0-t (ng.h/mL)	Ke. AUC 0-t (ng.h/mL)	Ct + Ke. AUC 0-t	Amount absorbed 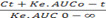	Cumulative amount absorbed (ng)	Fraction absorbed
0	0.00	0.00	0.00	0.00	0.00	0.00	0.00	0.00
0.5	1.67	0.42	0.42	0.05	1.72	0.01	0.01	0.00
1	17.06	4.68	5.10	0.56	17.62	0.06	0.07	0.01
1.5	53.54	17.65	22.75	2.50	56.04	0.19	0.25	0.05
2	81.08	33.65	56.41	6.20	87.28	0.29	0.55	0.11
3	101.54	91.31	147.71	16.25	117.79	0.40	0.94	0.18
4	155.30	128.42	276.14	30.37	185.68	0.62	1.57	0.30
6	136.43	291.73	567.87	62.47	198.89	0.67	2.24	0.43
8	121.31	257.74	825.61	90.82	212.13	0.71	2.95	0.57
10	109.68	231.00	1056.60	116.23	225.91	0.76	3.71	0.72
12	74.67	184.35	1240.95	136.50	211.17	0.71	4.42	0.85
24	23.80	590.78	1831.73	201.49	225.29	0.76	5.18	1.00

Ke = 0.11 h^-1^, AUC_0-∞_= 2702.89 ng/mL.h

## References

[1] Hartrick CT, Rozek RJ (2011). Tapentadol in pain management. A µ-opioid receptor agonist and noradrenaline reuptake inhibitor. CNS Drugs.

[2] Dewan Roshan Singh, Kusha Nag, Akshaya N TPHCl: A novel analgesic Saudi J Anaesth. 2013; 7(3): 322-326.

[3] Chen H, Mou D, Du D (2007). Hydrogel-thickened microemulsion for topical administration of drug molecule at an extremely low concentration. Int J Pharm.

[4] Alkilani AZ, McCrudden MT, Donnelly RF (2015). Transdermal Drug Delivery: Innovative Pharmaceutical Developments Based on Disruption of the Barrier Properties of the stratum corneum. Pharmaceutics.

[5] Prausnitz MR, Langer R (2008). Transdermal drug delivery. Nat Biotechnol.

[6] Richard C, Cassel S, Blanzat M (2020). Vesicular systems for dermal and transdermal drug delivery. RSC Adv.

[7] Liu L, Zhao W, Ma Q (2023). Functional nano-systems for transdermal drug delivery and skin therapy. Nanoscale Adv.

[8] Nilam H (2014). Patil, Padma V. Devarajan. Colloidal carriers for noninvasive delivery of insulin. Colloid and Interface Science in Pharmaceutical Research and Development. Elsevier.

[9] Azeem A, Khan ZI, Aqil M (2009). Microemulsions as a surrogate carrier for dermal drug delivery. Drug Dev Ind Pharm.

[10] Fouad SA, Basalious EB, El-Nabarawi MA (2013). Microemulsion and poloxamer microemulsion-based gel for sustained transdermal delivery of diclofenac epolamine using in-skin drug depot: *in vitro*/*in vivo* evaluation. Int J Pharm.

[11] Mishra R, Prabhavalkar KS, Bhatt LK (2016). Preparation, optimization, and evaluation of Zaltoprofen-loaded microemulsion and microemulsion-based gel for transdermal delivery. J Liposome Res.

[12] Ngawhirunpat T, Worachun N, Opanasopit P (2013). Cremophor RH40-PEG 400microemulsions as transdermal drug delivery carrier for ketoprofen. Pharm Dev Technol.

[13] Madikattu K (2016). Microemulsion based Transdermal Gels of Isradipine to Enhance Bioavailability: *In vitro* and *In vivo* evaluation. Asian Journal of Pharmaceutics.

[14] Smit JW, Oh C, Lannie C (2009). Effects of probenecid on tapentadol immediate release pharmacokinetics: Results of an open-label, crossover, drug-drug interaction study. J Clin Pharmacol.

[15] Lina Zhou, Xin Huang, Hongxun Hao (2022). Structure and pseudo-ternary phase diagram of water/Triton X-100/1-pentanol/cyclohexane microemulsion. Journal of Molecular Liquids.

[16] Moghimipour E, Salimi A, Leis F (2012). Preparation and evaluation of tretinoin microemulsion based on pseudo-ternary phase diagram. Adv Pharm Bull.

[17] Ujwala Shinde, Sharda Pokharkar, Sheela Modani Design and Evaluation of Microemulsion Gel System of Nadifloxacin Indian J Pharm Sci. 2012; 74(3): 237-247.

[18] Patel HK, Barot BS, Parejiya PB (2013). Topical delivery of clobetasol propionate loaded microemulsion based gel for effective treatment of vitiligo: *ex vivo* permeation and skin irritation studies. Colloids Surf B.

[19] Sintov AC, Shapiro L (2004). New microemulsion vehicle facilitates percutaneous penetration *in vitro* and cutaneous drug bioavailability *in vivo*. J. Control. Release.

[20] Ghosh P, Milewski M, Paudel K (2015). *In vitro*/*in vivo* correlations in transdermal product development. Therapeutic delivery.

[21] Mohammed D, Matts PJ, Hadgraft J (2014). *In vitro*-*in vivo* correlation in skin permeation. Pharmaceutical research.

[22] Draize J, Woodard G, Calvery H (1944). Methods for the study of irritation and toxicity of substances topically applied to skin and mucous membranes. J Pharmacol Exp Ther.

[23] Mrunali Patel R, Rashmin Patel B, Jolly Parikh R (2016). Novel isotretinoin microemulsion-based gel for targeted topical therapy of acne: formulation consideration, skin retention and skin irritation studies. Appl Nanosci.

[24] Binder L, Mazal J, Petz R (2019). The role of viscosity on skin penetration from cellulose ether-based hydrogels. Skin Res Technol.

[25] Yu YQ, Yang X, Wu XF (2021). Enhancing Permeation of Drug Molecules Across the Skin via Delivery in Nanocarriers: Novel Strategies for Effective Transdermal Applications. Front Bioeng Biotechnol.

[26] Boche M, Pokharkar V (2020). Microemulsion assisted transdermal delivery of a hydrophilic anti-osteoporotic drug: Formulation, *in vivo* pharmacokinetic studies, *in vitro* cell osteogenic activity. J Appl Pharm Sci.

[27] Patel P, Pol A, Kalaria D (2021). Microemulsion-based gel for the transdermal delivery of rasagiline mesylate: *In vitro* and *in vivo* assessment for Parkinson's therapy. Eur J Pharm Biopharm.

[28] Davanco MG, Campos DR, Carvalho PO (2020). *In vitro* - *In vivo* correlation in the development of oral drug formulation: A screenshot of the last two decades. Int J Pharm.

[29] Leanpolchareanchai J, Teeranachaideekul V (2023). Topical Microemulsions: Skin Irritation Potential and Anti-Inflammatory Effects of Herbal Substances. Pharmaceuticals (Basel).

[30] Pavoni L, Perinelli DR, Bonacucina G (2020). An Overview of Micro- and Nanoemulsions as Vehicles for Essential Oils: Formulation, Preparation and Stability. Nanomaterials.

[31] Shukla T, Upmanyu N, Agrawal M (2018). Biomedical applications of microemulsion through dermal and transdermal route. Biomed Pharmacother.

